# Responses to Mineral Supplementation and Salmon Lice (*Lepeophtheirus salmonis*) Infestation in Skin Layers of Atlantic Salmon (*Salmo salar* L.)

**DOI:** 10.3390/genes12040602

**Published:** 2021-04-19

**Authors:** Lene Sveen, Aleksei Krasnov, Gerrit Timmerhaus, Andrè Sture Bogevik

**Affiliations:** Nofima AS, Muninbakken 9, 9019 Tromsø, Norway; aleksei.krasnov@nofima.no (A.K.); gerrit.timmerhaus@nofima.no (G.T.); andre.bogevik@Nofima.no (A.S.B.)

**Keywords:** Atlantic salmon, salmon lice, minerals, mucous cells, transcriptomics, AI-model

## Abstract

The crustacean ectoparasite salmon louse (*Lepeophtheirus salmonis*), which severely affects Atlantic salmon health and welfare is one of the main problems of commercial aquaculture. In the present study, fish were fed a diet supplemented with extra minerals through the inclusion of a commercial additive (Biofeed Forte Salmon), substituting wheat in the control diet, before experimental infestation with salmon lice. Lice counts reduced with time but with no apparent effect of the diets. Further, fish fed the mineral diet had an overall higher number of blue (acidic) mucous cells, while the ratio of purple mucous cells was higher in the mineral diet. The transcriptional response in skin was enhanced at 7 dpc (copepodite life stage) in fish fed the mineral diet including immune and stress responses, while at 21 dpc (pre-adult life stage), the difference disappeared, or reversed with stronger induction in the control diet. Overall, 9.3% of the genes affected with lice also responded to the feed, with marked differences in outer (scale + epidermis) and inner (dermis) skin layers. A comparison of transcriptome data with five datasets from previous trials revealed common features and gene markers of responses to lice, stress, and mechanically induced wounds. Results suggested a prevalence of generic responses in wounded skin and lice-infected salmon.

## 1. Introduction

The salmon louse (*Lepeophtheirus salmonis*) is an ectoparasitic copepod causing large economical losses and fish welfare challenges in the Norwegian fish farming industry [1,2,3]. The lice feed on the mucus, skin, and blood causing skin ulceration and exhausting stress if left untreated [4]. The problems have escalated with increased commercial production of Atlantic salmon in sea cages. The parasite has developed resistance to orally administrated drugs, leading to a dramatic reduction in treatment efficacy. Improvement of salmon lice control with the use of new measures is one of the most active and rapidly expanding areas of aquaculture research. Promising solutions are reducing the contact of farmed salmon with salmon lice, such as lice skirts, lasers, cleaner fish, and snorkel cages reviewed in [5]. Integrated lice management also includes attempts of vaccine development [6,7], selective breeding [8,9], and functional feeds [10,11,12].

The salmon louse infects the skin of salmonid species where it feeds on the host skin, mucus, and blood [13]. The life cycle of the salmon louse includes eight stages, five larval stages (two naupliar stages, one copepodite stage, and two chalimus stages), and three post-larval stages (two preadult stages and one adult stage) [14,15]. The naupliar stages are not infective, while the larval copepodite stage attaches to the salmon and moults into the chalimus stages. The two pre-adult stages are fully motile and may jump between nearby hosts. In the last adult stage, fertilized females develop sets of egg strings, which upon hatching release a new generation of naupliar stages. While the infectious larval stages mainly feed on mucus and epidermal cells in proximity to the attachment site [16], blood-feeding and more excessive skin damage is observed in mobile pre-adult and adult lice stages [13,17]. The histopathological responses of the host towards the salmon lice copepodite are described with little tissue responses [18,19], followed by mild inflammation as the lice moult into pre-adult life stages [20]. The hosts transcriptional responses towards the salmon lice are on the other hand profound already at the copepodite stage with and large-scale changes in gene expression also rapidly occur in organs such as the spleen, liver and head kidney which suggests activation of systemic responses [21,22].

To the best of our knowledge, no studies are investigating the effect of mineral supplemented diets on salmon lice infestation in Atlantic salmon. The relative levels of naturally occurring and added trace minerals have changed due to transfer from marine to plant-based feeds [23], with largely unknown consequences for fish. The physiological importance of trace minerals in the modulation of immune response and overall disease prevention is well documented for humans and terrestrial animals [24,25,26]. Information in aquatic species is limited, but the basic metabolic functions of the trace minerals are believed similar across vertebrates [27]. Despite the recognition of the essential roles of minerals, little is known about the relationship between mineral nutrition and the resistance of fish to pathogens. Dietary levels adequate under a normal condition may become deficient or excessive when fish suffer from stress and diseases [28].

In this study, we wanted to examine the effect of the inclusion of a commercial feed additive (Biofeed Forte Salmon) with high mineral content, on the skin and immune responses towards salmon lice infestation in Atlantic salmon. The dietary effects on lice count were examined at multiple time points, while the hosts response to lice were assessed at 7 dpc (days post-challenge) and 21 dpc, with histological and transcriptomic tools [29,30]. We also performed meta-analyses to identify the repetitive patterns and markers of transcriptomic responses to the parasite in the skin of Atlantic salmon.

## 2. Materials and Methods

### 2.1. Diets

Two diets were produced at Aller Aqua Research (Büsum, Germany); control (C) diet and diet where wheat flour was substituted with Biofeed Forte Salmon additive (Biofeed AS, Trondheim, Norway); M-diet. Analysis of crude protein by the Kjeldahl method (*N* × 6.25) (ISO 5983-1997) and total fat according to Bligh and Dyer (1959) [31], showed similar contents of protein and fat in the diets. Content of ash (incineration at 550 °C ± 20 °C for 16 h, ISO 5984-2002) in the test feed (8.9%) was higher than in the control feed (5.8%), mainly due to acid-insoluble ash (ash not dissolved in boiling 3N HCL, mainly silicates), levels of iron, iodine and manganese were also increased (Table 1). The minerals in feed, whole fish and mucus were determined by inductively coupled plasma atomic emission spectroscopy (ISO 11885-1996). The mineral levels in the M-diet were below the upper limits allowed by regulations and close to levels in commercial feeds (e.g., Fe (110–300 mg/kg) and Mn (20–69 mg/kg) [32]. Iodine was at a higher level (still within regulations) compared to commercial feeds (0.1–9.6 mg/kg).

### 2.2. Lice Challenge Trial

**Figure 1:**
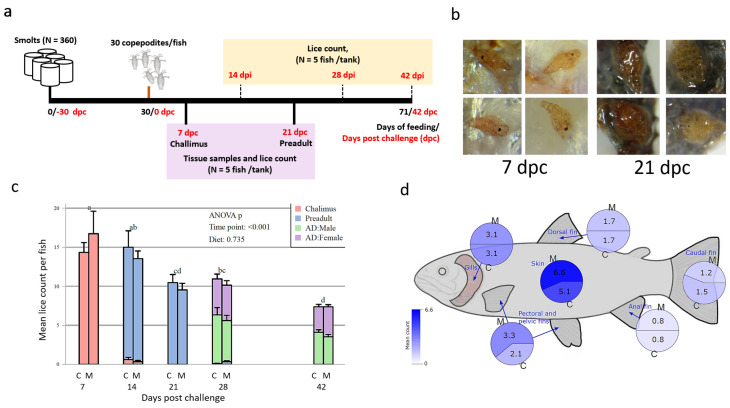
Experimental design and lice count (**a**) Experimental design. (**b**) Lice samples with chalimus (7 dpc) and preadult lice prior (21 dpc) to RNA extraction (**c**) Mean lice count (separated for the developmental stage of the lice) per fish at days post-challenge (dpc) for the two feeding groups. Two-way ANOVA for time points and diet, followed by Tukey post hoc test to identify differences between time points. Bars which do not share a letter are significantly different (*p* < 0.05) to each other. (**d**) Mean lice numbers at different body positions at 7 dpc, represented as pie charts for the two feeding groups. Colors from white to blue represent the scale from 0 to 6.6 (the maximum) lice per fish, for C and M diets.

In June 2019, 360 post-smolt Atlantic salmon (117 ± 1 g) were randomly distributed to six flow-through fiberglass tanks (400 L) at Stiftelsen Industrilaboratoriet (ILAB, Bergen, Norway). The fish had been smoltified in late May 2019 and kept at 25‰ salinity and 12 °C for 4 weeks prior to the start of the experiment. The fish were fed experimental feeds ad libitum for 71 days, with three tanks receiving the M-diet and three tanks receiving the C-diet. The feed intake was monitored the first 30 days of the experiment, but no dietary differences on feed intake or feed conversion ratio were observed (data not shown). The temperature was kept at 12 °C throughout the trial, while the salinity was 25–34‰ before the challenge with salmon lice, and 34‰ after the challenge. The fish in all tanks were infested with lice at day 30 by reducing the water levels in the tanks and adding salmon lice copepodites (strain LsGulen, reared at ILAB) at a concentration of 30 lice/fish. The tanks continued to receive the respective experimental feeds for another 41 days. All fish were weighed at the start, 1-day pre lice challenge, and at the termination of the trial 42 (dpc). At five time points during the trial (Figure 1), welfare skin scoring (ulcers, scale loss, etc.) according to the FishWell handbook [33], salmon lice counting, and sampling of mucus and skin were performed on five fish per tank (Supplementary Materials, page 1). Fish skin mucus was gently scraped off the surface using the blunt side of a sterile scalpel blade avoiding the skin sampling areas for histological and transcriptional analysis. The mucus was removed from the fish with a 1 mL pipette and transferred to 2 mL EppendorfTubes^®^ (Eppendorf, Hamburg, Germany) kept on dry ice during the sampling and stored at −80 °C. Four skin samples were collected from each fish. Intact skin was sampled from an area beneath the dorsal fin, above the lateral line and stored on RNAlater (Sigma, Saint-Louis, MO, USA) and 10% formalin CellStor Pot (CellPath, Mochdre, UK). Two skin samples with lice were collected and stored in the same manner. At 7 dpc, skin with lice was sampled from the ventral side between the pectoral and the anal fin. In the case of multiple lice, the site closest to the pelvic fin was selected. At 21 dpc lice in the ventral area was scarce, hence skin with lice was sampled in the dorsal region, between the dorsal fin and the base of the caudal fin. At the termination of the trial, egg strings were collected from adult lice females from 5 fish per tank. The egg strings were adjusted to a similar length (12 mm) prior to incubation in a flow-through system with seawater at 12 °C for 12 days. Hatched sea lice copepodites were counted.

### 2.3. Histology and AI-Model

Embedding, sectioning, and staining of the tissue samples were done at the Veterinary Institute in Harstad, Norway. For each fish, two tissue sections were processed: one sample with lice and one sample without lice excised from a standard area (dorsal part of the fish, under the dorsal fin, and above the lateral line). Tissue samples from the same fish were embedded and sectioned together. The tissue sections were hydrated in water and stained with 1% Alcian blue (Alfa Aesar) in 3% acetic acid for 15 min, transferred to 1% periodic acid (VWR) for 10 min, followed by Schiffs (Sigma-Aldrich^®^, Saint-Louis, MO, USA) reagent for 15 min, and finally for 30 s in hematoxylin (VWR, Radnor, PA, USA) before dehydration and mounting. AB/PAS staining stain mucous cells dark blue, purple or pink based on the acidity of the mucins [34]. AB/PAS-stained tissue sections of Atlantic salmon skin were scanned with an Aperio slide scanner (Leica, Microsystems Nussloch GmbH, Wetzlar, Germany). The digital AI-analysis was performed according to [19] for the skin tissue samples collected at 21 dpc. In total 36 randomly selected tissue samples (with and without lice), from 18 fish (*N* = 3 fish per tank) were analyzed with the AI-model.

### 2.4. Transcriptomics

Considering profound differences between the tissue compartments, we compared transcriptomes of the skin layers (epidermis + scale) and dermis in intact skin and attachment sites. All samples were examined and photographed prior to RNA isolation (Figure 1b), and the skin layers were separated under a stereoscope. Samples with and without lice were processed in the same manner. For the skin samples with lice, the lice were removed prior to RNA extraction, and five scales were picked directly beneath the feeding site and transferred to tubes with 400 µL lysis buffer (Qiagen, Hilden, Germany), and 20 µL proteinase K (50 mg/m). Further, all remaining scales were removed from the tissue sample. The section was trimmed to approximately 2 mm^2^, flipped to its basal side where all the muscle tissue and most of the subcutaneous adipose tissue was scraped with a sterile scalpel blade, leaving mainly the connective tissue. These samples were processed in the same way. In the figures, the outer skin layer containing epidermis and scales and an inner layer containing dermis are referred to as, respectively, OL and IL.

Infected and intact skin from five fish per treatment (C- and M- diet), at two time points (7 and 21 dpc), in total 80 samples were used for microarray analysis. Samples were randomly selected from all the six tanks, (*N* = 2 for two tanks and *N* = 1 for the remaining tank, a total of 5 fish per treatment).

Samples were homogenized in FastPrep 96 (MP Biomedicals, Eschwege, Germany) for 120 s at maximum shaking, then centrifuged and incubated at 37 °C for 30 min. RNA was extracted on Biomek 4000 robot using an Agencourt RNAdvance Tissue kit (Qiagen Norway, Oslo, Norway) according to the manufacturer’s instructions. RNA concentration was measured with NanoDrop™ One (Thermo Fisher Scientific, Waltham, MA USA) and quality was assessed with Agilent Bioanalyzer 2100. Samples with an RNA integrity number (RIN) of 8 or higher were accepted. Nofima’s 15 k Atlantic salmon DNA oligonucleotide microarrays SIQ-6 were manufactured by Agilent Technologies (Santa Clara, CA USA), and the reagents and equipment were purchased from the same provider. RNA amplification and labeling were performed with a One-Color Quick Amp Labeling Kit and a Gene Expression Hybridization kit was used for fragmentation of the labeled RNA. Total RNA input for each reaction was 200 ng. After overnight hybridization in an oven (17 h, 65 °C, rotation speed 0.01 g), arrays were washed with Gene Expression Wash Buffers 1 and 2 and scanned with an Agilent scanner. Bioinformatic package STARS [19] was used for data analyses. Global normalization was performed by equalizing the mean intensities of all microarrays. The individual values for each feature were divided by the mean value of all samples, thereby producing expression ratios (ER). The log2-ER values were calculated and normalized with locally weighted nonlinear regression (Lowess).

### 2.5. Data Analysis

Statistical analysis and data presentation were performed in R (version 3.5.2, https://www.r-project.org/, accessed on 1 October 2020) and MS Excel. ANOVAs and post hoc Tukey test were part of the stats-package (functions aov () and TukeyHSD ()). Differentially expressed genes were selected by criteria: expression ratio of >1.75-fold and *p* < 0.05 (*t*-test). Cluster and enrichment analyses of DEG were performed. The data was passed to the function hclust () (R stats package, for Euclidean distance and with complete linkage) and plotted with heatmap.2 () (gplots package). The dendrogram of the heatmap was cut into seven sub-clusters by the cutree () function (stats package). The number of sub-clusters was manually determined to optimize for distinctive expression profiles. Seven sub-clusters were identified, and group mean ER values for the groups and each cluster were plotted as bar plots with error bars (showing standard error of the mean, SEM). Functional annotations are manually curated in the microarrays database STARS. Over-representation of these categories within the seven clusters was analyzed by calculating Fisher’s exact tests (fisher.test () function with alternative hypothesis set to “greater”, stats package). A full list of DEGs is provided in Supplementary Materials, page 4.

## 3. Results and Discussion

### 3.1. Fish Feeding Trial and Lice Counts through the life Cycle of Salmon Lice

The salmon had an average body weight of 159 ± 7 g at Day 29 and 266 ± 4 g at Day 71 after start feeding the experimental diets, with no dietary effect observed (ANOVA, *p* = 0.94); (Supplementary Materials, page 2). The numbers of lice were counted at the stages of chalimus, pre-adult, adult males, and females on 5 fish per tank, and on all the remaining fish at the termination of the trial (Day 71; *n* = 21–25 fish/tank, Figure 1). At 7 dpc lice were distributed between the skin, gills and fins (Figure 1d), with mean lice values 16.7 for the C diet, and 14.3 for the M diet. Thereafter average counts were 15 chalimus lice/fish at 7 dpi, 10 preadult lice/fish at 21 dpi, and 8 adult lice/fish at 42 dpi (Figure 1). A significant reduction of lice numbers per fish was observed during the trial but there was no dietary effect (*p* = 0.74). At the end of the trial, 5 egg strings from each tank (15 per diet group) were hatched. The numbers of copepodites ranged from 773 to 845 per tank without significant difference between the dietary groups (*p* = 0.16), with an average of 822 and 793 copepodites per 5 egg strings from salmon fed with, respectively, C and M-diet (Supplementary Materials, page 3).

### 3.2. Increased Iodine Content in Fish Mucus and Number of Acidic Mucous Cells in the Skin of Fish Fed Mineral Enhanced Diet

Mineral salts account for up to 1% of the mucus mass affecting its secretion and rheological properties [35,36], hence we were interested in the effect of the M-diet on fish mucus. Because the amount of mucus sampled from one fish (100–400 µL) was relatively small for mineral analysis, preference was given to one trace element—iodine. The iodine levels in the mucus and body of salmon fed on the M-diet were nearly three times higher than those of the C-diet (*P* < 0.01, Table 2), reflecting the iodine content in the diets. The role of iodine in fish is known mainly in relation to thyroid hormones, which regulate metabolic activity [37]. In mammals, the major portion of iodine is concentrated in the thyroid gland, while non-hormonal iodine is found in different tissues including salivary glands, eye, gastric mucosa, and cervix, where its functions are not well known [38]. Hence, at this point, we can only speculate about the function of iodine in fish mucus.

**Figure 2:**
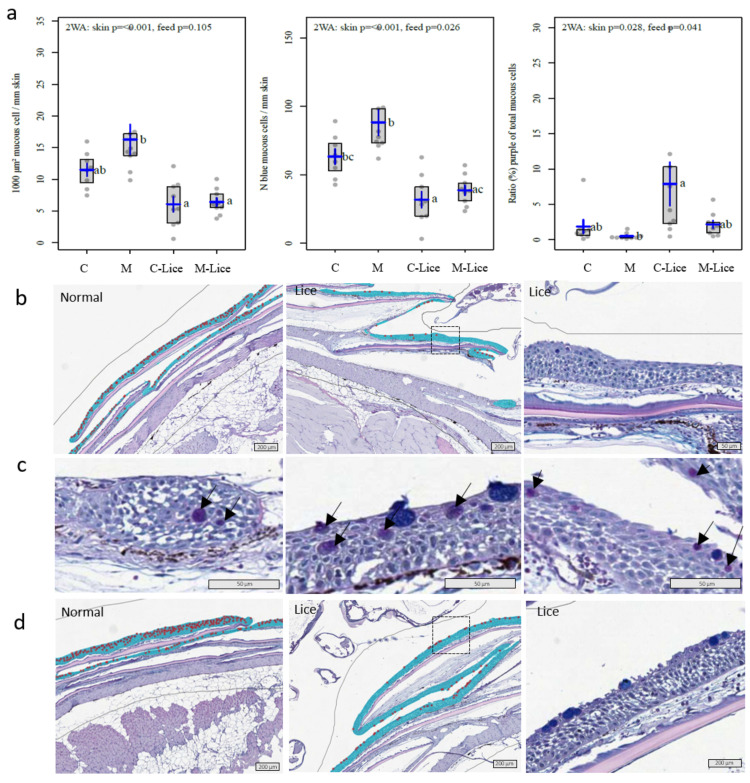
Mucous cell parameters and histology (**a**) Mucous cell area, mucous cell number per mm of skin, and the ratio of purple to total (blue + purple) mucous cell count. Two-way ANOVA followed with Tukey post hoc test, *p*-values for feed (normal skin control diet, versus normal skin mineral diet (M)) and skin (normal skin vs. lice affected skin) are shown on top of the plots. Boxes, which do not share a letter are significantly different (*p* < 0.05). (**b**) AB/PAS stained tissue sections of fish skin fed the control diet. First column normal skin, second column skin with lice, third column enlargement of the dotted area in the second column. (**c**) Purple mucous cells (arrow) on skin tissue sections with lice fed the control diet. (**d**) Similar to b for the mineral diet. Artificial color overlay as presented by the AI-model, blue epidermis, red circles represent mucous cells counted as objects. Tissue sections without the artificial color overlay are presented for the illustration of mucous cell color (panel b), and epidermal morphology (vascularization and rough appearance of epithelial surface cells) typically observed at the feeding site (third column a and c).

Overall, the number of blue (acidic) mucous cells per mm of skin was higher (*p* = 0.026, Two-way ANOVA) in fish fed the M-diet compared to the C-diet at 21 dpc (Figure 2). The number of mucous cells at the feeding site was lower compared to skin samples without lice in both dietary treatments. In our recent work, comparing Atlantic salmon skin at six different body positions, we found no effect of body position on the number of blue mucous cells/mm skin or mucous cell area per area of skin, while the number of purple mucous cells was higher in the anterior region of the fish compared to the other five positions [29]. Hence, the observed reduction in mucous cell number is likely due to lice attachment and not variations due to body position. A reduction of mucous cell number at the feeding site has also been observed in coho salmon (*Oncorhynchus kisutch*), and sockeye salmon (*Oncorhynchus nerka*) [39], and Atlantic salmon [11]. The observed decrease in mucous cell density at the feeding site may be due to mechanical disruption of the tissue caused by the ectoparasite.

Further, the ratio of neutral mucous cells (purple) to the total number of mucous cells (pink + blue) was highest in samples with lice from fish fed with the C-diet. In healthy Atlantic salmon skin, the dominance of glycans with negative charges results in blue staining of the mucous cells with the AB/PAS staining technique [34,40]. Our results indicate a possible link between the dietary mineral level, the acidity of the mucins, and response towards lice. Relevant to these findings, it was recently demonstrated that stress-induced skin mucin O- glycosylation changes in Atlantic salmon, particularly in fish subjected to both chronic stress and an acute challenge test [41]. To the best of our knowledge, there is no literature on mucin glycosylation concerning the mineral nutrition in fish or lice infestation. Since host mucin glycosylation governs interactions with pathogens [40,42], this could be an interesting cue to follow.

### 3.3. Mineral Supplementation Enhances the Transcriptomic Responses towards Salmon Lice

**Figure 3:**
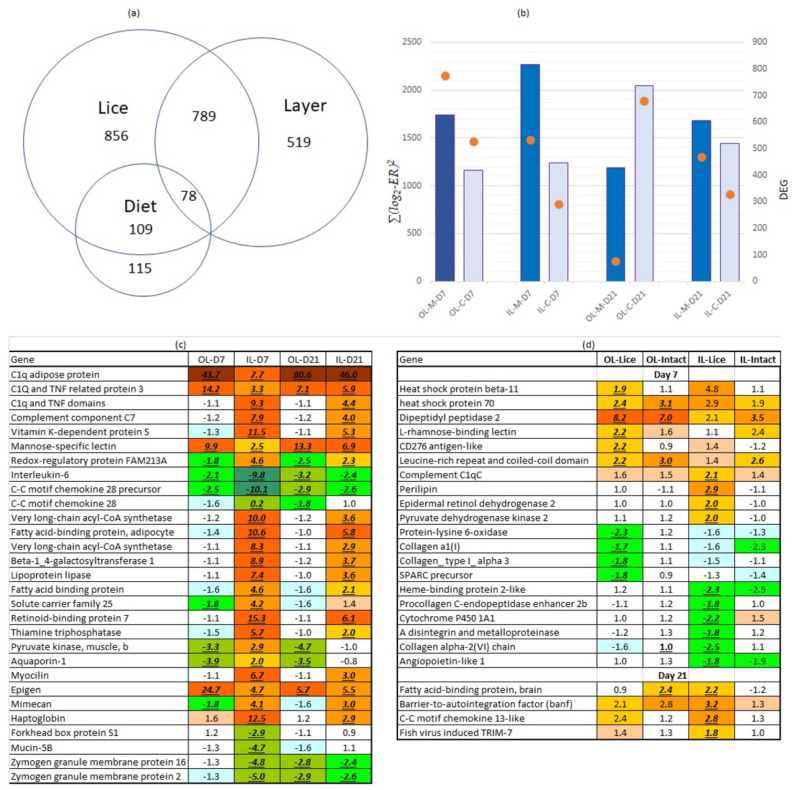
Summary of transcriptome analyses (**a**) Venn diagram: numbers of genes that showed responses to lice, the difference between the skin layers, and diets in at least one comparison. (**b**) The magnitude of responses to lice assessed as ∑(log_2_-ER)^2^ (bars, left axis) and numbers of DEG (orange circles, right axis). (**c**) Genes with stronger responses to salmon louse in outer and inner skin layer at 7 dpc and 21 dpc. Data are expression ratios (folds) to intact skin (means for both diets), significant differences are indicated with underlined italic bold. (**d**) Effect of mineral supplementation on gene expression in outer and inner skin layer 7 dpc and 21 dpc. Data are expression ratios (folds) of salmon fed with M- and C-diets in lice infected (L) and intact skin (means for both diets), significant differences are indicated with underlined italic bold. Abbreviations: OL, IL—outer and inner layers, M, C—diets, D—days post-challenge. Red and green color in (c) and (d) denote up and down-regulation of the genes.

Transcriptome analyses with 15 k Atlantic salmon DNA oligonucleotide microarray examined the relationship between responses to lice from one side, localization of transcripts in skin layers, and effects of mineral supplementation from another. Eight study groups (2 feeds (M- and C-diet) × 2 skin layers (outer and inner) × 2 sample types (intact and infested skin) were included in analyses. Of the 1827 DEG that responded to infestation with lice, 44% showed a higher abundance of transcripts in either the outer or inner layer, and 9.3% were different between M- and C-diet (Figure 3). The scales of expression changes were similar at the two time-points (7 and 21 dpc) and in both skin layers (Figure 3). The higher number of DEG in salmon fed with M-diet suggested that mineral supplementation enhanced the responses to the parasite at 7 dpc. Two weeks later difference disappeared in the inner skin layer (dermis) and reversed in the outer skin layer (epidermis + scale) with a higher number of DEG in the C-diet.

**Figure 4:**
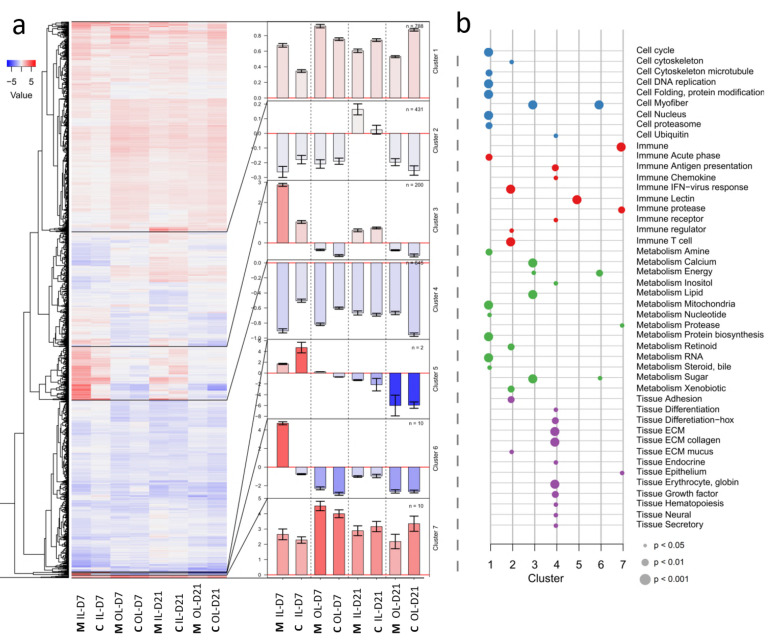
Transcriptomic responses to lice and diets at 7 and 21 dpc (**a**) Heatmap of mean log2-ER with dendrogram for the genes. One line represents one gene, and each column is a study group. Blue is for downregulation and red for upregulation. The heatmap was cut into seven clusters. The bar plots in the middle show the mean values of the respective clusters with +/− SEM error bars. Black lines in the heatmap indicate where clusters begin and end. The numbers of genes per cluster are shown. (**b**) The enrichment analysis of functional categories of STARS (Krasnov et al., 2011a) was performed within the clusters. Each vertical line represents one cluster. Dots on these lines indicate a significant enrichment of the categories shown on the right end (Fisher test *p*-value < 0.05). Colors indicate categories related to Cell structures and processes, Immune system, Metabolism and Tissue structure and development. Abbreviations: OL, IL—outer and inner layers, M, C—diets, D—days post-challenge (dpc).

To obtain a general overview of the transcriptomic responses to the lice in the two different diets, and in the two skin layers, the DEG were divided into seven clusters by expression profiles (Figure 4). The majority of DEG in cluster 1 were constantly up-regulated in infected skin and contained genes involved in various cellular and metabolic processes. In this cluster, the magnitude of response was higher in the M -diet at 7 dpc, whereas the response was reversed 21 dpc being higher in the C-diet. Down-regulation prevailed in clusters 2, with few differences between treatments. An exception was a higher response in the inner skin layer in fish fed the M-diet at 21 dpc, with genes related to immunity, metabolism, mucus, and cell adhesion. Cluster 3 and 6 showed a strong difference between the diets in the inner skin layer at 7 dpc. Genes in these clusters were mainly related to myofiber proteins and motor activity, and in cluster 3 also to metabolism. More than 30 genes encoding myofiber proteins showed >10-fold change at either at 7 dpc or 21 dpc, being the gene class most responsive to the M-diet (Supplementary Materials, page 4). A similar induction of myofiber proteins as a response to salmon lice infection has been found in previous experiments [22], the opposite response with massive down-regulation of myofiber proteins has also been observed [43]. At the present we can only speculate about the function of myofibers in salmon skin, which is waiting for exploration. Cluster 4 was enriched in down-regulated genes associated with tissue structures and processes with no apparent dietary effects. A hallmark of cluster 5 were lectins with high transcription in the inner skin layer of the C-diet at 7 dpc. In cluster 7, immune genes and extracellular proteases were induced by lice at both time points.

Further, we looked closer at individual genes (Figure 3). The M-diet affected gene expression in the skin at the feeding site, and also intact skin, and a slight stimulation of stress and immune responses were observed in both layers of the skin. At 7 dpc upregulation was shown by two heat shock proteins, rhamnose binding lectin, a skin-specific cd276-like gene, uncharacterized dipeptidyl peptidase, and lrrcc1 and c1qc1 complement component. Most of these genes have unknown roles, but we have observed their involvement in immune and stress responses in many transcriptome studies with Atlantic salmon. At 21 dpc the M-diet weakened the suppression of several immune genes. At the same time, a panel of genes encoding collagens, enzymes, and other regulators involved in deposition and maturation of extracellular matrix were down-regulated in the M-diet after challenge. Cyp1a1, a key enzyme of xenobiotic biotransformation has shown downregulation in Atlantic salmon under various inflammatory conditions.

The parasite also stimulated a suite of genes (519) with marked specificity to the different skin layers (Figure 3). In addition to gene classes mentioned in the previous paragraph, it is worth noting several members of the c1q-like family and mannose-specific lectin which showed stronger induction in the outer skin layer. The complement system was more active in the inner skin layer as well as lipid metabolism and several genes from other metabolic pathways. Mimecan and epigen are involved in the differentiation of connective tissue and epidermal structures [44,45]. Up-regulation of myocilin, a viscous component of body fluids, and concurrent downregulation of mucin-5b, zg2, zg16, il6, and chemokines suggested changes in the composition of mucus. Of note is that these genes showed layer-specific differences only at 7 dpc.

### 3.4. Gene Markers of Responses to Lice

**Figure 5:**
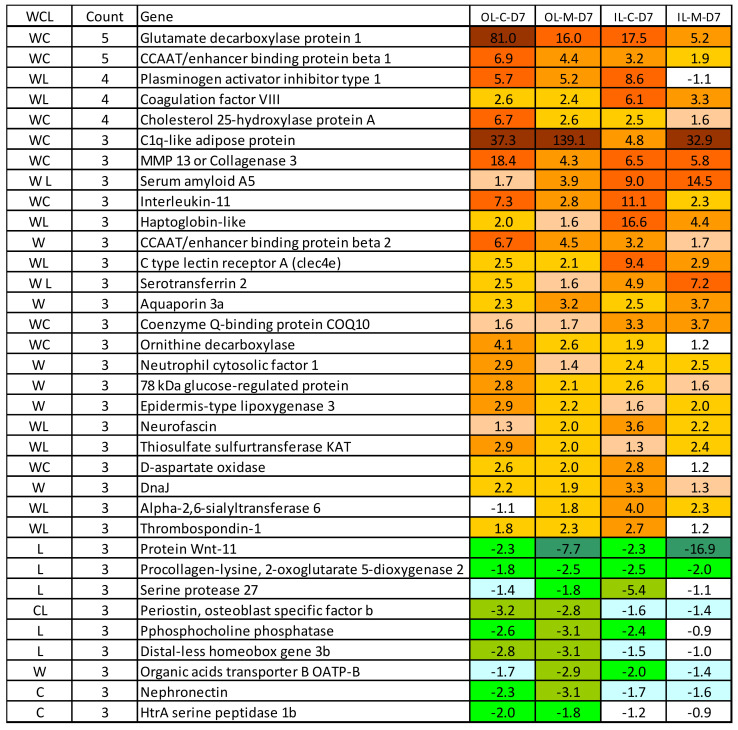
Gene markers of lice responses to lice in Atlantic salmon skin, data are folds to intact skin. Genes with expression differences in at least three of five data sets were selected. First column represent study with response, only stable responses for wound (W), cortisol (C) and difference between the skin layers (L). Second column—number of data sets with differential expression (maximum five). The last columns show the transcriptional response in the present study. Abbreviations: OL, IL—outer and inner layers, M, C—diets, D—days post-challenge (dpc). Red and green color denote up and down-regulation of the genes.

In our experience, transcriptome responses to lice in different trials show lower stability and greater variation in comparison with bacterial and especially viral challenges. The identification of stable gene markers characteristic for lice infestation has the potential to improve our knowledge of lice and host interaction. Here, we searched for gene markers of responses to lice across five datasets representing independent studies. Three data sets were from our published experiments with lice infestation: skin responses at different lice stages [21], responses to lice, cortisol implants and their combination [43], and effects of sexual maturation and sex hormones applied as feed additives [46]. As we have observed activation of wound healing pathways as host response during lice infestation, we also included our recent trial investigating the transcriptomic response to deep mechanically induced wounds in post-smolts [47,48]. The analysis identified 34 markers of lice infestation (Figure 5). Importantly, almost all the markers were activated in process of wound healing, while almost half of them responded to the administration of cortisol without the presence of lice. Hence, it is likely that the degree of injury inflicted by the lice, and general stress responses are the key factors influencing host responses towards the lice.

The most stable responses were shown by cebpb, a multifunctional transcription factor regulating diverse stress and immune processes, and gad1, a putative enzyme of glutamate metabolism involved in the biosynthesis of neurotransmitters [49]. Three genes were present in four of five data sets: two components of the blood coagulation cascade (pai-1 and fviii), and ch25 h, an enzyme of cholesterol metabolism involved in various immune processes [50,51] and known for strong responses to viruses in Atlantic salmon [52]. The highest upregulation in this study was observed in emblematic markers of inflammation including matrix metalloproteinase mmp13, cytokine il11, acute-phase protein saa5 and one of multiple hemoglobin binding haptoglobins (hp). The role of the most induced gene for c1q-like protein is unknown, but members of this large fish-specific multigene family exhibit very high immune activity in Atlantic salmon, as seen from our transcriptome data. Lice infestation consistently up-regulates several immune effectors, stress gene (chaperone) grp78, and genes involved in metabolism and control of differentiation. The number of down-regulated genes was smaller and most of them are with roles in various developmental processes. Three genes were down-regulated with cortisol and only oatp-b, an organic anion transporter with broad specificity, responded to wound healing. Other highly responsive genes have not been characterized and their role in lice infestation awaiting further investigation.

## 4. Conclusions

Until present, dietary manipulations have resulted in a limited reduction in the numbers of lice at best, but research in this area will continue. Here, we show that mineral supplementation results in an overall higher number of acidic mucous cells, and enhances the host transcriptional response towards salmon lice, with a marked difference between the skin layers. We have further verified that wound healing responses play a key role in the salmon lice interactions. Stimulation of healing can be recommended as a guideline for the development of functional feeds.

## Figures and Tables

**Table 1:** Ingredient composition, and macro nutrients and minerals in the feeds.

Ingredients	Control Feed (C)	Mineral Feed (M)
Marine ingredients	30.5	30.5
Plant ingredients	66.2	62.2
Micronutrients	3.3	3.3
Biofeed Forte salmon	0.0	4.0
Macro nutrients and minerals		
Crude protein (g/100 g)	47.7	46.9
Total fat (g/100 g)	23.7	23.5
Water (%)	3.5	3.7
Dry matter (%)	96.5	96.3
Ash (%)	5.8	8.9
Acid-insoluble ash (%)	0.03	1.2
Iron (Fe; mg/kg)	110	270
Arsenic (As; mg/kg)	2.7	2.5
Selenium (Se; mg/kg)	0.7	0.7
Zink (Zn; mg/kg)	140	140
Iodine (I; mg/kg)	6.0	17.0
Manganese (Mn; mg/kg)	25.0	30.0

**Table 2:** Iodine content in the whole fish, the feeds, and skin mucus.

Diet	Days	Source	Iodine (mg/kg)	Ratio M/C Diet
Control		Feed	6.0	
Mineral		Feed	17.0	2.8
	0	Whole fish	0.26 ± 0.06	
Control	71	Whole fish	0.34 ± 0.03	
Mineral	71	Whole fish	0.75 ± 0.05	2.2
Control	71	Skin mucus	0.16 ± 0.02	
Mineral	71	Skin mucus	0.48 ± 0.06	3.3

## Data Availability

Microarray data were submitted to NCBI Geo Omnibus (pending accession).

## Supplementary Materials

The following are available online at https://www.mdpi.com/article/10.3390/genes12040602/s1, Supplementary Materials.
